# Migraine and Patent Foramen Ovale: Is Platelet Hyperactivity the Underlying Mechanism?∗

**DOI:** 10.1016/j.jacbts.2022.04.001

**Published:** 2022-06-27

**Authors:** Robert J. Sommer, Barbara T. Robbins

**Affiliations:** Department of Medicine, Columbia University Irving Medical Center, New York, New York, USA

**Keywords:** migraine with aura, oxidative stress, patent foramen oval, platelets, tissue factor

**Figure:**
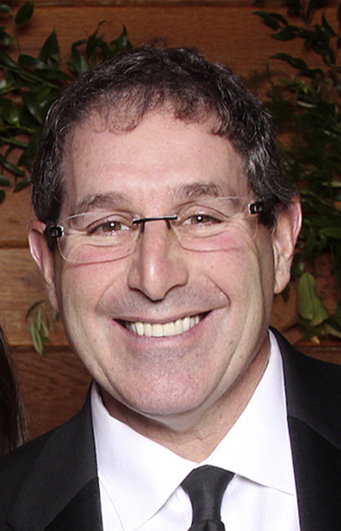

Migraine headache (MHA) is a multifactorial clinical syndrome with a substantial societal impact. It is the second leading cause of disability in the United States, with a worldwide prevalence of nearly 15%.^1^ However, the underlying biology of MHA remains poorly understood. As a result, the clinical response to the growing number of migraine treatments is unpredictable from patient to patient.

Patent foramen ovale (PFO), a remnant of the fetal heart circulation, has been linked to MHA for more than 2 decades. At least 25% of the MHA population, like the general population, has a PFO. It is clear from innumerable observational series that in some PFO-MHA patients, the associated right-to-left shunt is mechanistically involved with the MHA symptoms because PFO closure eliminates or substantially ameliorates symptoms. On the other hand, for other MHA patients, the presence of a PFO may be completely incidental. The inability to distinguish one from the other has been a critical roadblock to proving the benefit of PFO closure or even to proving a definitive biologic relationship between PFO and MHA.

In this issue of *JACC: Basic to Translational Science*, Trabattoni et al^2^ from Milan present an extremely elegant and sophisticated laboratory analysis of platelet function in patients with PFO and MHA. Briefly, using flow cytometry and other methodologies, they were able to demonstrate a unique hyperactive platelet state in this population when compared with healthy control individuals. This hyperactivity was characterized by twice the normal platelet membrane expression of tissue factor and phosphatidylserine, resulting in an increased endogenous thrombin potential, but without a corresponding increase in the classical markers of platelet activation such as P-selectin and activated glycoprotein Idibia. In addition, compared with control individuals, the PFO-MHA patients demonstrated twice the serum macrovesicle concentration, a marker for cellular activation, as well as a state of oxidative stress, as demonstrated by reduced glutathione levels and significantly higher levels of intracellular platelet reactive oxygen species. Trabattoni et al^2^ then demonstrated that with the administration of clopidogrel or with subsequent transcatheter closure of the PFO, the markers of platelet hyperactivity returned to control levels and coincided with MHA cessation.

The findings of Trabattoni et al,^2^ and even the terminology itself, are far above the paygrade of the average neurologist or cardiologist. However, the implications of this research are profound. The study, which is the first to reveal a biologic link between PFO and MHA, may not only change the way we evaluate people with MHA in the future but may also fundamentally change the study of MHA as a disease state, giving support to the proposed idea that MHA symptoms may be the common clinical expression of multiple distinct underlying pathophysiologic mechanisms, of which the PFO is one.

The association between PFO and MHA dates to the 1990s, when transcatheter PFO closure for recurrent stroke prevention was beginning on a large scale. Following PFO closure, some patients with a history of MHA reported a significant reduction or complete elimination of their headaches. However, despite similar observations from cardiologists all over the world, the initial group of prospective PFO-MHA studies was unable to prove that closing the PFO was beneficial. In these trials, some of the study patients had dramatic reduction of headache frequency and severity, but that benefit was not statistically different from that seen in the control individuals. In part, this was attributable to the small cohort sizes and to the large placebo effect seen in most MHA medication trials. However, perhaps the more important issue, unrecognized at the time, was the fact that the presence of a PFO did not necessarily indicate a causal link to the MHA. Most migraineurs do not have a PFO, implying that there must be other, “non–PFO-related” MHA mechanisms. However, because PFO is so common in the population, there are sure to be migraineurs with non–PFO-related MHA mechanisms who also have a PFO. Because the early trialists had no way to distinguish the patients with the “causal” PFOs from those with “incidental” PFOs, and because closing an incidental PFO, by definition, will have no effect on MHA symptoms, these trials had no chance of succeeding without a method for qualifying only the “causal” PFOs into the study cohort.

In a recent open-label observational series from Columbia University,^3^ it was demonstrated that in the PFO-MHA population, treatment with thienopyridines (platelet P2Y_12_ receptor inhibitors) substantially reduced or eliminated MHA symptoms in up to two-thirds of patients. It was speculated that platelet aggregation or platelet activation byproducts from the venous circulation, rather than being filtered in the lungs, were crossing the PFO to reach the brain in supraphysiologic levels, triggering the MHA. That notion was reinforced by the observation that the MHA response to P2Y_12_ platelet inhibition correlated nearly perfectly with the MHA response to subsequent PFO closure. In principle (and in recent practice), the study investigators proposed that the response to a trial of P2Y_12_ inhibition medication could be used to screen for the “causal” PFOs. This concept will be rigorously tested as part of the Gore RELIEF (GORE CARDIOFORM Septal Occluder Migraine Clinical Study) clinical study,^4^ set to resume enrollment later this year.

However, this clinical strategy has significant limitations. For patients with chronic daily headache or high-frequency episodic migraine, the clinical response to P2Y_12_ inhibition will be easily recognizable over a short period of time. However, for patients with substantial variability of MHA frequency from month to month, as well as for those with severe but less frequent symptoms, this approach is much less practical. In addition, the open-label response to medication treatment is complicated to assess, because of both the purely subjective nature of MHA and the large placebo effect associated with any MHA therapy. Finally, although these blood-thinning medications are very safe in the age group most affected by migraine, they are not entirely without risks.

At its core, the work of Trabattoni et al^2^ reproduces, at the cellular level, exactly what was suggested in the Columbia clinical study^3^: that either P2Y_12_ inhibition or PFO closure eliminates headaches, in a majority of these patients, by changing the patients’ biology.

These stirring new laboratory data themselves have limitations. Principally, because Trabattoni et al^2^ studied the platelets of only patients with both PFO and MHA, there is no way to discern whether the platelet hyperactivity identified is unique to this group or whether it may be present in all migraineurs or in all patients with PFO. Furthermore, we do not yet know if the platelet assessment of Trabattoni et al can help distinguish the “causal” PFOs from the “incidental” PFOs or if additional refinements to the platelet testing will be needed. Future studies could compare the platelet activity of control patients who have neither PFO nor MHA with those of patients with PFO only, those with migraine only, those with both PFO and MHA who respond to P2Y_12_ agents, and those with both who do not respond to platelet inhibition. If we were able to reproducibly demonstrate differences in platelet activity in the PFO-MHA patients who respond to P2Y_12_ drugs, we would have the first set of biomarkers capable of directing personalized therapy in the MHA population. In addition, the measurable, objective nature of these laboratory findings would potentially eliminate the need for administering P2Y_12_-inhibiting drugs, the bleeding risks associated with administering those drugs, and the potential confounder of any placebo benefit reported by the patients.

The other major roadblock to using these findings in clinical practice is that the laboratory testing described by Trabattoni et al^2^ is highly specialized and not generally available in a normal hematology laboratory. Shipping samples to a specialized laboratory is also problematic because the testing described requires fresh blood samples. However, if the platelet hyperactivity in the PFO-MHA patients is proven to be a marker for successful therapy by subsequent research, we could begin a search for more accessible biomarkers, leading to blood tests that could ultimately be ordered by any physician treating MHA patients.

This work opens an unprecedented opportunity in the MHA space, one that will require a complex interdisciplinary collaboration between hematologists, headache neurologists, and interventional cardiologists. Over the next few years, in the United States, nearly 2,000 migraineurs will be enrolled in the Gore RELIEF clinical study. Within this study, some MHA patients with PFO will respond to P2Y_12_ inhibition and some will not, and some will not have a PFO at all. If we could identify U.S. laboratories at RELIEF study sites (or at least in the same cities) that are capable of reproducing the Italian platelet studies, the fundamental question about the specificity of the platelet hyperactivity could be answered. This is a rallying cry.

## Funding Support and Author Disclosures

Dr Sommer has a relationship with W.L. Gore & Associates; is the National Cardiology primary investigator on the Gore RELIEF clinical study; was the national coprimary investigator on the ASSURED study; and is currently an investigator in the REDUCE-PAS study. W.L. Gore & Associates provides institutional funding to Columbia University. Dr Robbins has reported that she has no relationships relevant to the contents of this paper to disclose.
